# YOLOv12n-RMB: An Improved Dual Target Recognition Network for Seedling Maize and Field Weeds in Cold Regions

**DOI:** 10.3390/plants15182876

**Published:** 2026-09-20

**Authors:** Jinyang Li, Yagang Du, Xianbin Wu, Hongfei Dai, Chuntao Yu, Ziyi Zhu, Wei Zhang

**Affiliations:** 1 College of Information and Electrical Engineering, Heilongjiang Bayi Agricultural University, Daqing 163319, China; ljy970118@163.com; 2 Heilongjiang Academy of Land Reclamation Sciences, Harbin 150036, China; 3 College of Engineering, Heilongjiang Bayi Agricultural University, Daqing 163319, China; 4 College of Agriculture, Heilongjiang Bayi Agricultural University, Daqing 163319, China

**Keywords:** maize, weed identification, RGB image, YOLOv12n-RMB, multi-frequency feature aggregation, bidirectional weighted fusion

## Abstract

Accurate identification of maize seedlings and weeds at the seedling stage in dry farmland of cold regions serves as an essential prerequisite for field variable weeding and intelligent plant protection operations. Existing YOLO networks exhibit prominent limitations in feature extraction for fine weeds that grow close to the ground and feature fusion across different scales, and show poor adaptability to complex field conditions including illumination fluctuations, tiny weed interference, and mixed soil textures. To address these issues, this study focuses on maize fields in the cold region at a high latitude in Heilongjiang and collects RGB field images of maize plants at the two to five leaf stage. Using YOLOv12n as the baseline model, the internal convolutional blocks within the C3k2 structure are replaced with RepViTBlock to construct the C3k2-R module. The Multi-Frequency in Multi-Scale Attention (MFMSA) module is embedded into A2C2f to obtain A2C2f-M. BiFPN is adopted to replace the original concatenation block to form Concat-B. By integrating the above three optimized modules, the improved model YOLOv12n-RMB is developed. The results demonstrate that YOLOv12n-RMB achieves an AP@0.5 of 0.9745 for maize and 0.7820 for weeds, with an overall mAP@0.5 improvement of 0.0421 relative to the original YOLOv12n. The proposed model can perform robustly under diverse field working conditions such as strong light, weak light, and varying seedling ages. This research provides visual technical support for field weed density evaluation and intelligent decision-making for variable weeding equipment.

## 1. Introduction

Maize is a staple food crop in the dry farming system of northern China. As one of the major maize-producing regions in China, Heilongjiang Province lies in a cold region at high latitude with limited accumulated temperature and short periods without frost, and maize seedlings exhibit weak stress resistance at the seedling stage. Field weeds germinate simultaneously with maize plants, competing for light, water, and soil nutrients while inducing pests and diseases, which directly causes maize yield loss and quality degradation [1,2]. Conventional uniform herbicide spraying across entire fields leads to excessive pesticide application, aggravating agricultural pollution from diffuse sources and ecological pressure on black soil [3]. Under such circumstances, variable precision weeding has become a key direction for green maize plant protection in cold regions. Rapid and accurate visual identification of crop seedlings and field weeds serves as a core technical foundation for realizing variable pesticide application and intelligent weeding operations.

Traditional machine vision relies on manually extracted color and texture features combined with classical classification algorithms to complete target recognition. However, handcrafted features show weak noise resistance and are prone to missed detection of small targets and false detection from background clutter in complex field environments [4,5]. Deep learning can automatically mine deep image features and achieves substantially better recognition accuracy and generalization ability than traditional vision methods [6]. Among existing models, the YOLO series has become a mainstream framework for fast on-site detection of seedlings and weeds in farmland, owing to its fast inference speed and flexible architecture. Nevertheless, targeted optimization is still required for specific farmland scenarios [7,8]. Different YOLO variants present distinct trade-offs between accuracy and computational cost, and appropriate selection should be made according to actual field deployment requirements [9].

Most existing improvement schemes are developed for mature weeds, and fail to sufficiently capture weak texture features of tiny herbaceous weeds growing close to the ground at the seedling stage [10]. Conventional multi-scale fusion via simple concatenation tends to dilute shallow weed features and leads to low recall rates [11]. Numerous accuracy-focused improvement schemes simultaneously increase model parameter count, which limits their adaptability to mobile terminals with limited computing power [12]. To balance accuracy and inference overhead, existing studies adopt lightweight approaches such as depthwise separable convolution, model pruning, and knowledge distillation for network compression [13]. Meanwhile, Ghost and reparameterized bottleneck structures are introduced to optimize backbone feature extraction [14]. YOLOv8 and YOLOv9 improve field detection performance by simplifying the neck structure and reconstructing bottleneck modules, respectively [15,16]. Building upon YOLOv9, a real-time framework incorporating a hybrid EfficientViT backbone with cascaded group attention has demonstrated robust detection under occlusion, illumination variations, and visual similarity against complex backgrounds [17]. YOLO11 integrates C3k2 and attention modules and achieves balanced overall performance, yet it still suffers from feature loss when facing extremely small seedling-stage weeds [18]. The core goal of lightweight network design is to reduce computational cost with controllable accuracy loss, to support deployment on embedded devices [19].

Studies on multi-scale optimization have verified that bidirectional weighted pyramids and attention modules can strengthen interaction between shallow and deep features, and suppress false detections caused by soil and crop residues [20,21,22]. The idea of frequency domain differential processing also provides new insights for target discrimination under complex field backgrounds [23]. Collaborative optimization of multi-scale structures and attention mechanisms can simultaneously enhance the representation capacity of target contours and semantic information [24]. Most existing detection studies focus on warm-climate farmlands and greenhouses, while few dedicated algorithms have been developed for dry ridge farmland in Heilongjiang at high latitude [25]. Seedling-stage fields in cold regions suffer from highly variable illumination and a high proportion of bare soil. Motion blur is easily introduced during handheld image acquisition, which leads to obvious performance degradation when general models are transferred to these scenarios [26]. Seedling weeds and crops share highly similar morphological features at the seedling stage, making recognition far more challenging than at the middle and late crop growth stages [27]. Although unmanned aerial vehicle remote sensing detection achieves wide coverage, it delivers insufficient accuracy for tiny weed targets [28]. Most current improved networks increase computational overhead while boosting detection accuracy, which prevents stable, fast on-site inference on low-power computing devices. Balancing small target accuracy and inference efficiency remains a critical challenge to be addressed for field weed detection [29].

A comprehensive review of current studies on weed identification reveals that mainstream detection networks face three core gaps: weak feature extraction capability for tiny weeds, oversimplified multi-scale feature fusion strategies, and limited ability to distinguish high- and low-frequency background noise in field environments. To address these shortcomings and adapt to the field imaging conditions of maize grown on ridges in high-latitude regions of Heilongjiang, this study uses an RGB dataset of maize seedlings and weeds at the two to five leaf stage, collected by a DJI Action3 camera. YOLOv12n is optimized from three perspectives: backbone feature extraction, frequency domain enhancement, and weighted feature fusion. The RepViTBlock, MFMSA, and BiFPN modules are integrated to construct the YOLOv12n-RMB detection model. Multiple comparative experiments and on-site field validation tests are carried out to achieve robust identification of seedling maize and weeds. This work provides technical support for variable precision weeding and intelligent plant protection operations in cold regions.

## 2. Materials and Methods

### 2.1. Data Collection and Dataset Construction

**Figure 1:**
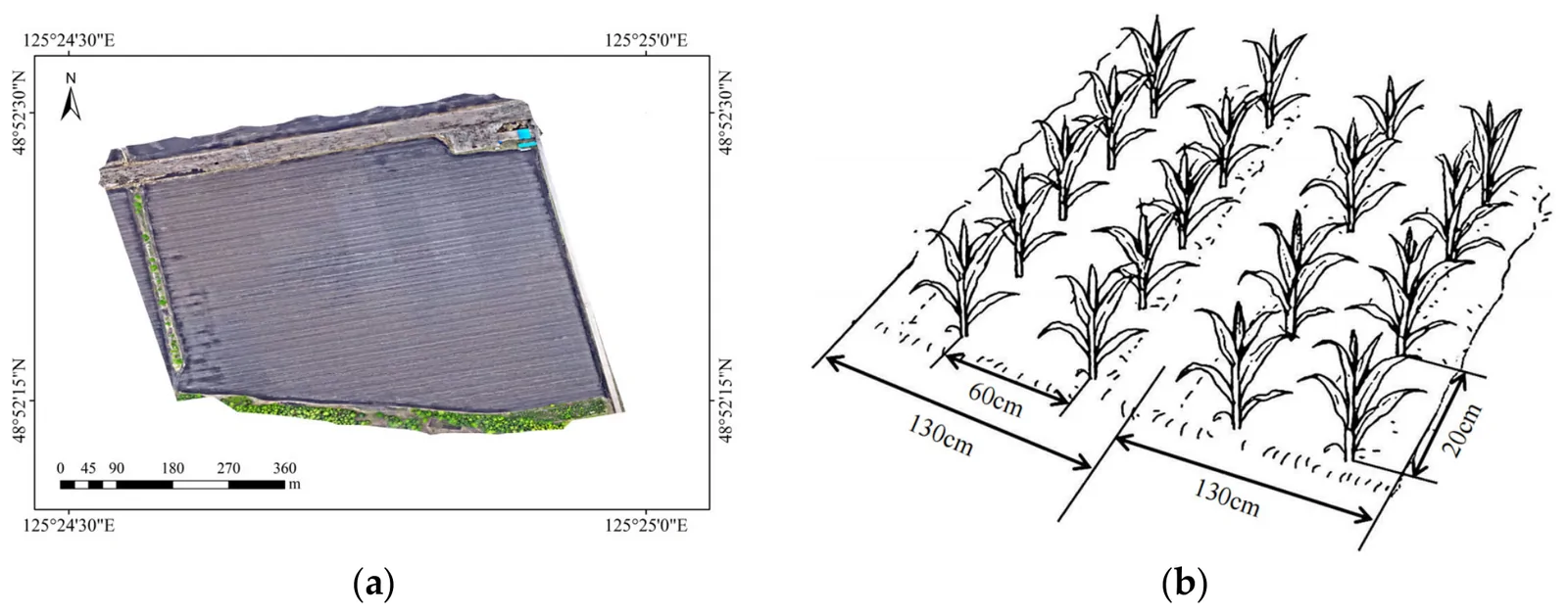
Overview of the experimental area. (**a**) Geographical location of the experimental field in Nenjiang County, Heilongjiang Province, China; (**b**) Schematic of the 130 cm ridge double row planting pattern with 60 cm row spacing and 20 cm plant spacing.

The experiment was conducted at Jianshan Farm, under the Jiusan Administration Bureau in Nenjiang County, Heihe City, Heilongjiang Province, China. This region has a cold temperate humid monsoon climate, with an annual average temperature of 0.4 °C, roughly 2500 h of annual sunshine, average annual precipitation of around 500 mm, and a frost-free period of about 118 days. It is located in the fourth and fifth accumulated temperature zones, with an effective accumulated temperature of roughly 2200 °C, making it a typical dry farming area at high latitude with limited heat. The experimental field uses a double row sowing pattern on 130 cm-wide ridges. Two rows of maize are evenly planted on each ridge platform, with a row spacing of 60 cm and plant spacing of 20 cm. This planting arrangement makes efficient use of ridge surface space, supports more uniform maize population distribution, and improves light energy utilization efficiency. The overview of the experimental area is shown in Figure 1.

**Figure 2:**
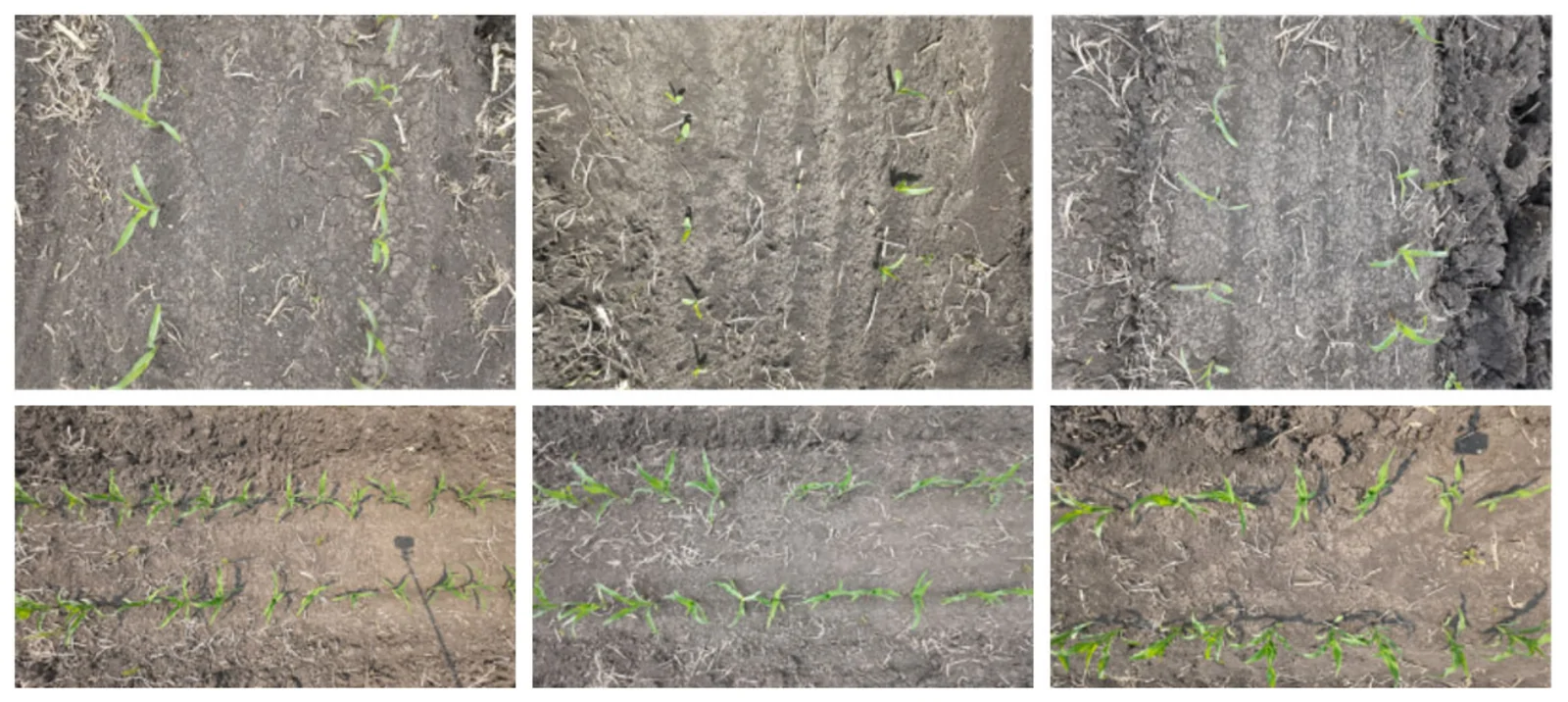
Representative original field images acquired by DJI Action3 camera at 40 to 60 cm height during the maize 2 to 5 leaf stage, covering diverse illumination and weed growth conditions. These samples are selected from the full dataset of *n* = 1000 original images.

Field images were captured between 10 May and 30 May 2026, using the DJI Action3 camera (SZ DJI Technology Co., Ltd., Shenzhen, China) during the 2 to 5 leaf stage of maize. This growth stage was selected because it corresponds to the critical weeding operation window in maize production, when weed competition with seedlings is most intense and variable-rate herbicide application or mechanical weeding is typically conducted. Two image resolutions were adopted, namely 4000 × 3000 pixels and 4000 × 2250 pixels. Both resolutions were retained in the dataset and used for subsequent training, validation, and testing. Prior to network input, all images were uniformly resized to the standard input size of the model, so images of either original resolution were processed through the same pipeline and the model could perform stable recognition on both. The camera height above the ground ranged from 40 to 60 cm during shooting. To construct a representative dataset that can fully reflect complex maize field environments, image acquisition closely focused on practical field challenges. Diverse scenarios covering different illumination conditions, weed growth stages, background complexity, spatial distribution complexity, and weed species were included in the dataset to lay a data foundation for improving model robustness. Overexposed, blurred, and other low-quality images were eliminated from the collected samples, and 1000 original images were finally obtained. Figure 2 shows several representative original field images.

LabelImg Version 1.8.6 software was applied for data annotation, and annotation files were saved in XML format. Two target categories, including maize and weed, were labeled within each image. To avoid data leakage between different subsets, the 1000 original images were first divided into training set, validation set, and test set at a ratio of 8:1:1, yielding 800, 100, and 100 images, respectively. Data augmentation was subsequently applied only to the training set so as to increase image quantity and diversity, while the validation and test sets retained original images without any transformation.

Augmentation operations were divided into two parts. The first part was geometric augmentation. One operation was randomly selected each time from horizontal flip, vertical flip, 180-degree rotation, 90-degree clockwise rotation, and 90-degree counterclockwise rotation. These transformations synchronously modified the coordinate values of YOLO label boxes. The second part was color augmentation. Each augmented image underwent transformations for brightness (0.75 to 1.25), contrast (0.80 to 1.20), saturation (0.85 to 1.15), and sharpness (0.80 to 1.30). The selection of these operations and parameter ranges was determined according to the imaging characteristics of field maize seedlings. Geometric transformations including flips and rotations were adopted because seedlings and weeds present arbitrary orientations under handheld shooting, and these operations simulate varied viewing angles without distorting target morphology. The color parameter ranges were set with reference to agricultural image augmentation studies and verified by preliminary experiments. The brightness range of 0.75 to 1.25 covers typical weak light and strong sunlight conditions in the field, while the contrast, saturation, and sharpness ranges were kept within moderate intervals to prevent color distortion and maintain the natural appearance of augmented images.

During augmentation, for each original training image, one geometric operation was first randomly selected, and the bounding box coordinates were synchronously adjusted, followed by color augmentation with randomly sampled parameter values within the specified ranges. This combined procedure was repeated to generate multiple augmented variants per original image. A total of 2000 augmented images were produced from the 800 original training images. Together with the 800 original images, the training set comprised 2800 images. The final training, validation and test sets contained 2800, 100 and 100 images respectively, constituting a total of 3000 images.

### 2.2. YOLOv12 Object Detection Model

YOLOv12 is a one-stage object detection framework. Its core architecture consists of an input layer, backbone, neck, detection head, and output layer. With a series of integrated architectural innovations, YOLOv12 meets the demands of high real-time performance and low computational cost in practical application scenarios [30]. YOLOv12 integrates a regional attention module, which divides feature regions through a joint optimization strategy combining spatial grouped convolution and channel recalibration. This design improves detection accuracy while preserving a large receptive field [31]. To alleviate gradient blocking issues in deep networks, YOLOv12 uses residual efficient aggregation structures, which reconstruct feature aggregation paths to build bottleneck units and lower computational overhead. At the architectural level, YOLOv12 eliminates redundant position encoding, optimizes the MLP channel ratio, and adopts large separable convolution to strengthen spatial information modeling capacity. For more efficient memory access, YOLOv12 increases inference speed by reducing redundant stacked blocks [32]. YOLOv12n is selected as the baseline model in this study, as it delivers the best tradeoff between accuracy and efficiency across the full YOLOv12 series.

### 2.3. Design of Improved Network YOLOv12n-RMB

Maize seedling field images present inherent detection challenges: extremely small weed targets, high textural similarity between weeds and bare soil, and unbalanced fusion of features across different scales. Despite its strong performance, YOLOv12n still has notable gaps in three aspects relevant to this scenario: local feature extraction, frequency domain feature discrimination, and cross-scale feature fusion. The standard C3k2 unit only extracts local features through stacked conventional convolutions, and lacks sufficient capacity to capture weak edge textures of tiny weeds. The original A2C2f feature enhancement unit does not differentiate high-frequency weed details from low-frequency background information, so valid high-frequency weed features are easily overwhelmed by large-area low-frequency noise from soil. The original network directly concatenates multi-scale features via the Concat operation without an adaptive weight allocation mechanism, so shallow features of small weeds tend to be diluted by deep large-scale maize features.

**Figure 3:**
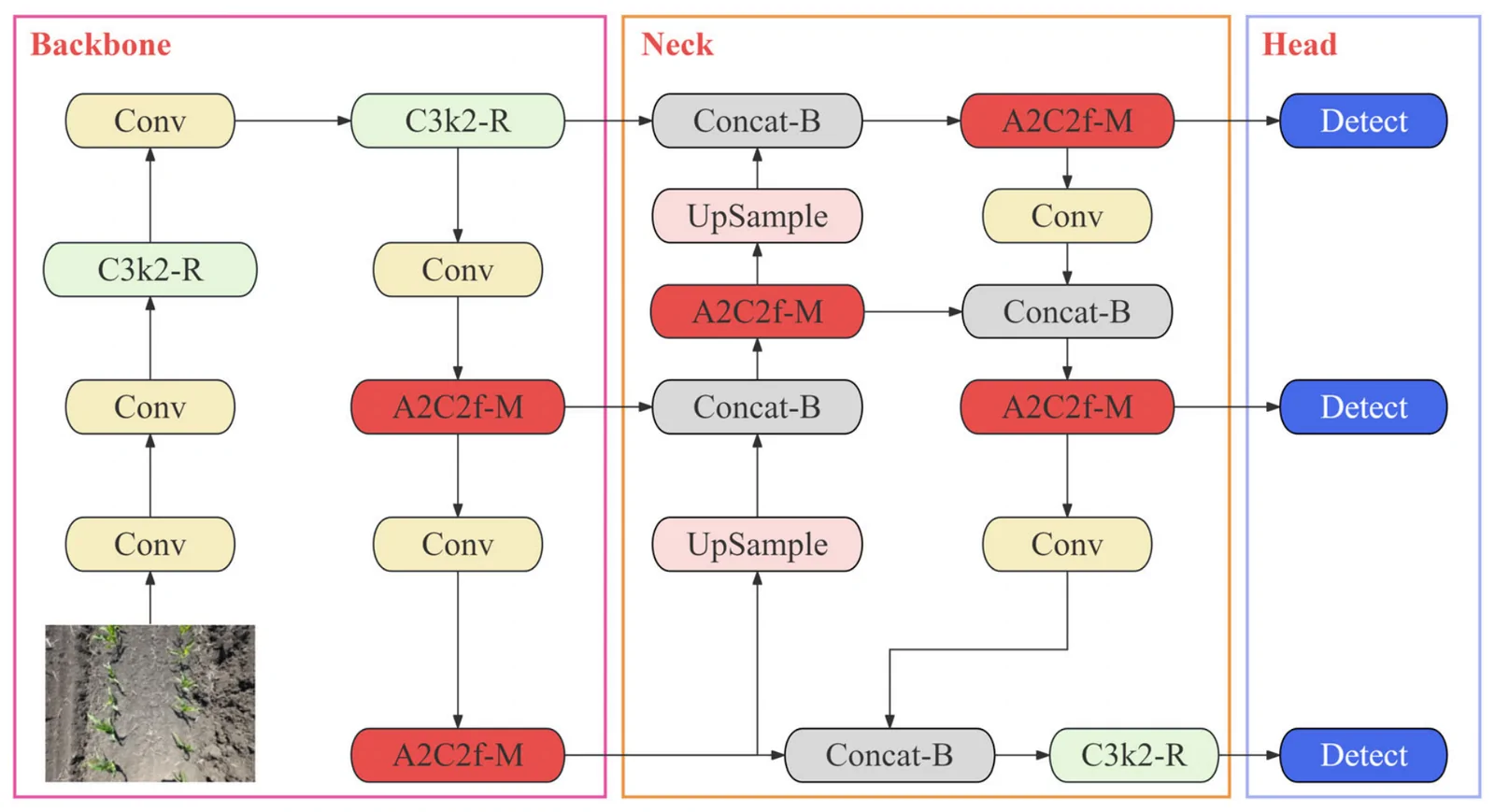
Network architecture of YOLOv12n-RMB, showing the Input, Backbone, Neck, and Head components with the C3k2-R, A2C2f-M, and Concat-B improved modules.

Accordingly, this study optimizes YOLOv12n along three dimensions: backbone feature extraction, multi-frequency multi-scale feature enhancement, and cross-layer weighted feature fusion. Three structures, namely RepViTBlock, MFMSA, and BiFPN, are incorporated in sequence to build the proposed YOLOv12n-RMB detection model, as illustrated in Figure 3.

#### 2.3.1. C3k2-R Module

RGB field images collected in this scenario are characterized by tiny weed target sizes, low contrast between weeds and backgrounds consisting of bare soil and crop straw, as well as texture noise introduced by motion blur. The original C3k2 module extracts features only through stacked ordinary serial convolutions and lacks effective mechanisms for cross-channel information interaction. Weak texture features of tiny weeds are easily lost during deep multi-layer convolution propagation, which significantly reduces weed recognition accuracy. RepViTBlock uses reparameterized multi-branch convolutions and dual-path feature processing across spatial and channel dimensions [33]. It enhances fine-grained feature extraction capacity for tiny targets through decoupling of training and inference structures. Therefore, this study replaces the original internal convolutional feature unit of C3k2 with RepViTBlock to reconstruct the feature extraction unit C3k2-R.

**Figure 4:**
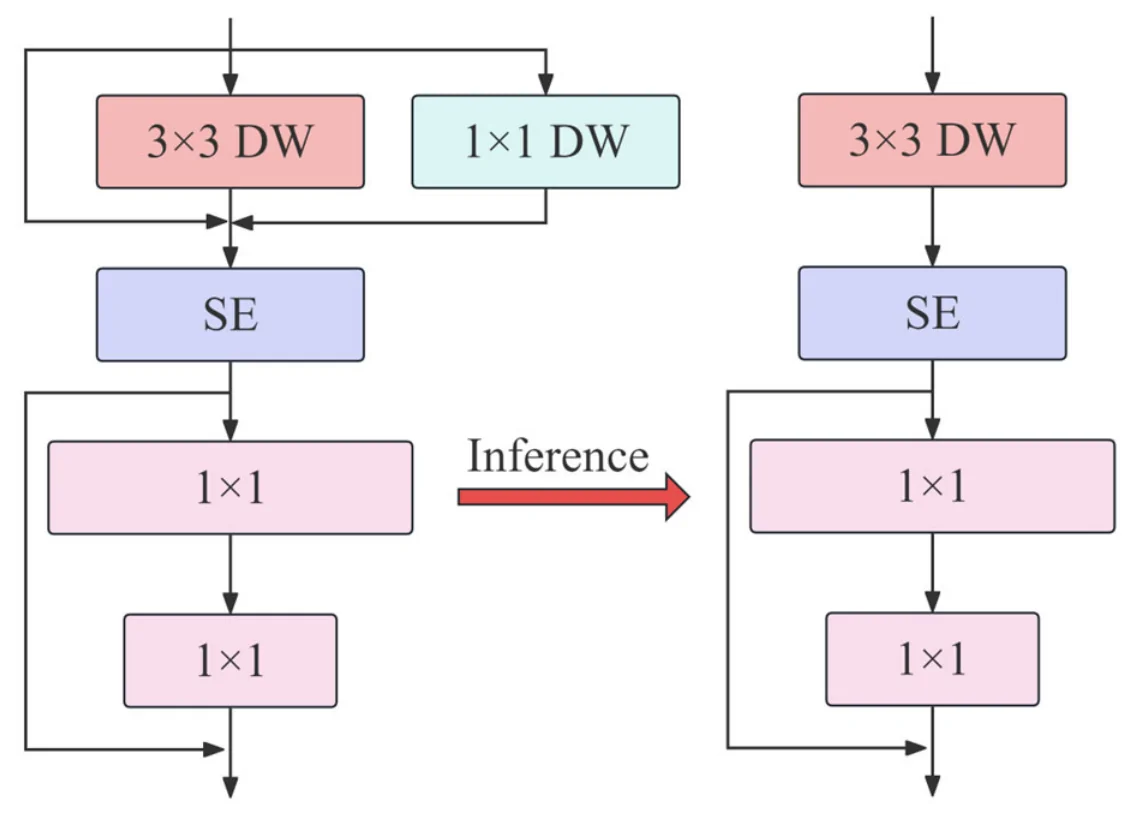
Structure of the RepViTBlock module, consisting of a Token Mixer with reparameterized depthwise convolution (RepVGGDW) and Squeeze-and-Excitation (SE) attention, followed by a Channel Mixer with residual connection.

The proposed C3k2-R block fully retains the two-branch split and residual connection bottleneck architecture of the original C3k2 unit. Only the internal serial convolution feature extraction unit is replaced by RepViTBlock. The structure of the RepViTBlock module is shown in Figure 4. Through joint optimization of the parallel spatial and channel dual pathways, RepViTBlock enhances the propagation efficiency of tiny weed features across network layers and improves the model’s ability to capture small, fine-grained field targets. RepViTBlock splits the feature processing pipeline into dedicated Token Mixer and Channel Mixer components to perform spatial feature mining and cross-channel interaction enhancement, respectively. The Token Mixer is purpose-built for spatial dimension feature extraction, and comprises the reparameterized depthwise convolution RepVGGDW and the Squeeze-and-Excitation (SE) channel attention module. RepVGGDW arranges three parallel branches, including 3 × 3 depthwise convolution, 1 × 1 depthwise convolution, and identity mapping. The 3 × 3 convolution captures large-range spatial textures. The 1 × 1 convolution supplements local fine edge features of weeds. The identity branch preserves original shallow features. Independent convolution operations for each channel help constrain model parameters and optimize spatial feature extraction performance for tiny targets. The SE channel attention block aggregates global channel statistical information via global average pooling, and adaptively learns non-linear weight relationships across channels. It amplifies channel responses for valid weed features and suppresses channel weights associated with soil background noise to improve feature discrimination between foreground targets and background clutter.

The Channel Mixer is responsible for multi-channel feature interaction. It first expands the channel dimension of features output from the Token Mixer, doubling the channel count to fully capture composite texture differences between weeds and maize plants. Convolutional operations are then used to compress features back to the original channel dimension, to screen and refine the most discriminative features. Finally, a residual connection is added between the module output and original input to fuse shallow local details and deep global semantic information.

#### 2.3.2. A2C2f-M Module

The original A2C2f feature enhancement unit in YOLOv12n performs feature filtering only using convolutions of uniform scale, and does not apply differentiated processing to different frequency components in input images. Useful recognition features for field weeds are concentrated in high-frequency details, including fine leaf edges and slender stems. Bare soil and intact maize plants, by contrast, represent low-frequency background contour information. Since the original module lacks dedicated separation mechanisms for high- and low-frequency components, high-frequency weed details are easily lost, and low-frequency soil noise continuously interferes with detection results. The proposed MFMSA module first decomposes input features in the frequency domain to separate high-frequency detail components from low-frequency background components. Two sets of independent multi-scale convolutions are then used to enhance features of the two components separately. Cross-scale attention is further applied to achieve adaptive aggregation of multi-scale features, which selectively preserves fine weed edges while suppressing soil background noise [34]. Accordingly, this study embeds the MFMSA module into the internal feature transmission branch of A2C2f to build the improved A2C2f-M feature enhancement unit.

**Figure 5:**
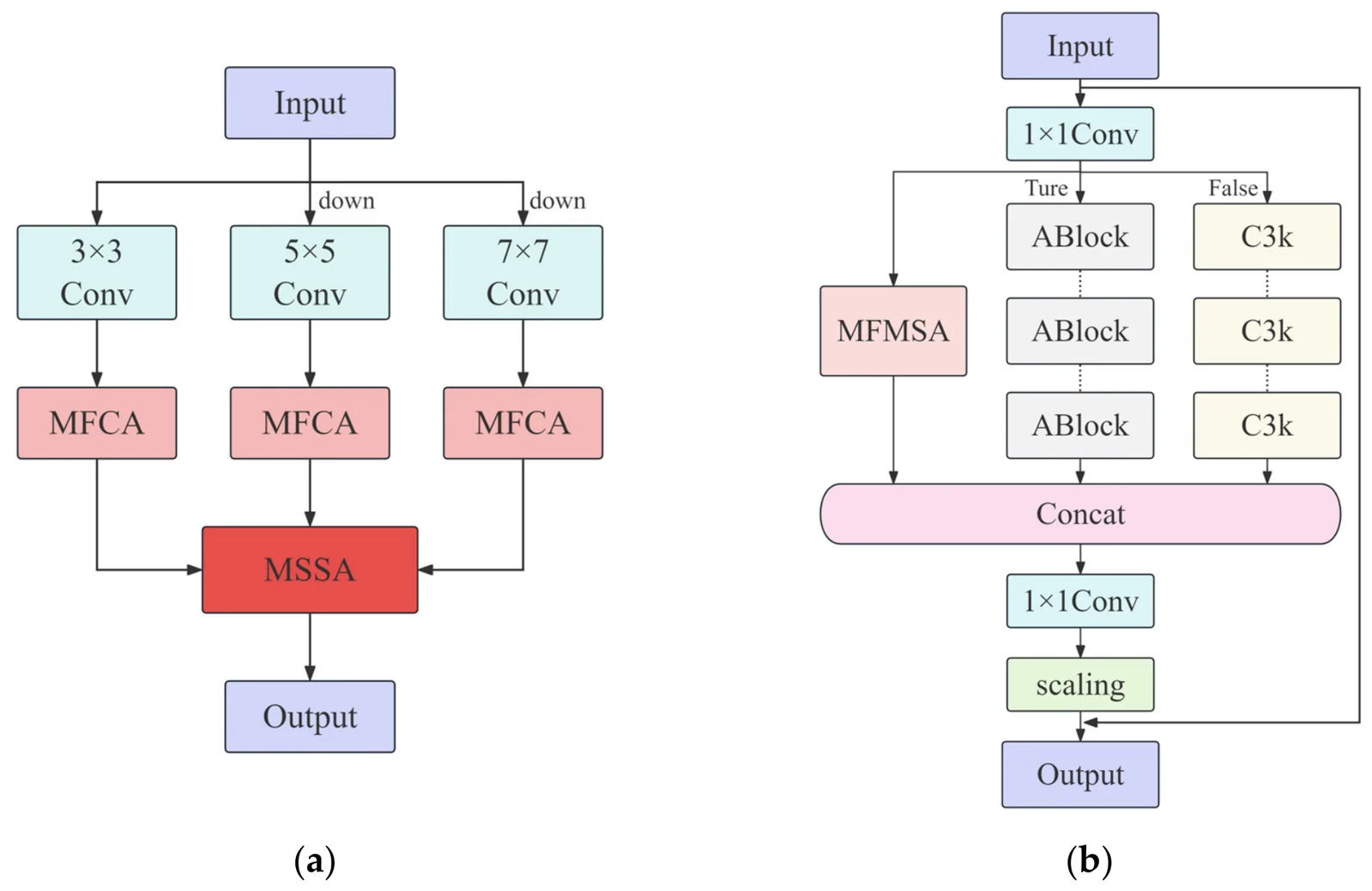
Structure of the A2C2f-M module. (**a**) Multi-frequency in Multi-Scale Attention (MFMSA) with Discrete Cosine Transform (DCT) based frequency decomposition, multi-frequency channel attention (MFCA), and multi-scale spatial attention (MSSA); (**b**) A2C2f-M constructed by embedding MFMSA into the A2C2f unit.

The proposed A2C2f-M retains the core framework of the original A2C2f. After the input features go through channel compression by the basic convolution layer of A2C2f, feature enhancement is completed through three sequential processing steps. The input feature map is first split into S scale branches. Each branch adjusts channel counts via downsampling and 1 × 1 convolution to balance multi-scale feature coverage and computational efficiency, as defined in Equation (1).

(1) $$C_{s}=\max(\frac{C}{\gamma^{s-1}},C_{\min}),\gamma =\frac{1}{2},$$

where *C* denotes the channel number of the input feature map; *γ* represents the channel reduction ratio with γ equal to 0.5; C_min_ stands for the minimum value of channel number. The structure of the A2C2f-M module is shown in Figure 5.

Multi-frequency channel attention (MFCA) transforms the feature map of each scale branch into a frequency-domain representation via DCT. The feature map is calculated as a weighted sum of different frequency basis images, as shown in Equation (2).

(2) $$X_{i}^{s,k}=\sum_{h,\omega }{\left(X_{i}^{s}\right)}_{:,h,\omega }D_{h,\omega }^{u_{k},\nu_{k}},$$

where $X_{i}^{s,k}$ denotes the k-th frequency component of the feature map from the s-th scale branch; *h*,*w* represent the spatial index of the feature map; *u_k_*_,_*v_k_* stand for the frequency index.

The basis images are generated by cosine functions, as shown in Equation (3).

(3) $$D_{h,\omega }^{u_{k},\nu_{k}}=\cos\left(\frac{\pi h}{H_{s}}(u_{k}+\frac{1}{2})\right)\cos\left(\frac{\pi \omega }{W_{s}}(\nu_{k}+\frac{1}{2})\right),$$

where *H_S_* denotes the height of the feature map for this scale branch; *W_S_* represents the width of the feature map for this scale branch.

Frequency statistics are compressed through global average pooling, max pooling, and min pooling. A channel attention map is generated via two fully connected layers to calibrate the feature map and suppress noise channels, as shown in Equation (4).

(4) $$\overset{\wedge }{X_{i}^{s}}=X_{i}^{s}\times M_{i}^{s},$$

where $M_{i}^{s}$ denotes the channel attention map; $X_{i}^{s}$ represents the original feature map.

Multi-scale spatial attention (MSSA) generates foreground attention maps and background attention maps for calibrated features through 1 × 1 convolution. Learnable parameters are combined to strengthen the response of target regions, as shown in Equations (5)–(7).

(5) $$F_{i}^{s}=\sigma (Conv2D_{1}(\overset{\wedge }{X_{i}^{s}})),$$

(6) $$B_{i}^{s}=1-F_{i}^{s},$$

(7) $${\overset{-}{X}}_{i}^{s}=Conv2D_{3}(\alpha_{i}^{s}(\overset{\wedge }{X_{i}^{s}}\times F_{i}^{s})+\beta_{i}^{s}(\overset{\wedge }{X_{i}^{s}}\times B_{i}^{s})),$$

where $F_{i}^{s}$ denotes the foreground attention map; $B_{i}^{s}$ represents the background attention map; $\alpha_{i}^{s}$ and $\beta_{i}^{s}$ stand for learnable parameters, which control the information flow weights of foreground and background, respectively.

Features from each scale branch are first aggregated after resolution alignment via upsampling. Element-wise addition is performed with the identity connection features. Convolutional operations are then applied to restore channel dimensions, and the final enhanced features are output.

#### 2.3.3. Concat-B Module

**Figure 6:**
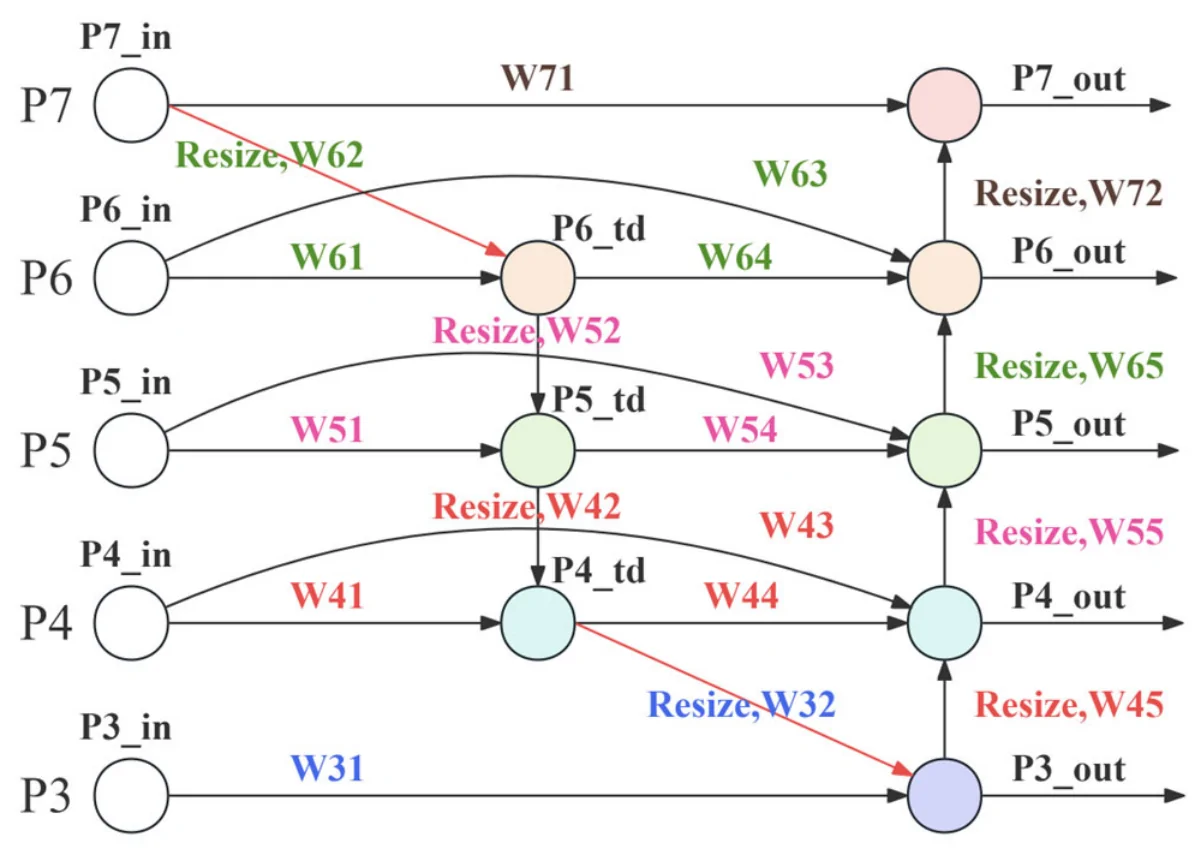
Principle of BiFPN, showing P3 to P7 multi-scale feature maps with top-down and bottom up weighted fusion paths. Colored circles represent operators, and uncolored circles represent intermediate features.

YOLOv12n uses the basic Concat operation to complete channel concatenation of features from different network layers. This operation performs undifferentiated channel stacking for multi-scale features, without accounting for the varying information contribution of features from different layers. Shallow features have high resolution and carry rich fine details of tiny weeds, but receive relatively low weight during direct concatenation. Deep features, by contrast, capture global semantic contours of large maize targets. This undifferentiated fusion dilutes valid shallow weed features, ultimately leading to a high missed detection rate for tiny weeds. To address this limitation, BiFPN constructs bidirectional feature transmission paths that combine top-down and bottom-up pathways. Learnable normalized weighting coefficients are assigned to features of different scales to adaptively amplify the feature weights of shallow small targets and balance information across multi-scale features [35]. The principle of BiFPN is illustrated in Figure 6.

This study replaces the original simple Concat concatenation with BiFPN weighted fusion logic and constructs the Concat-B module for multi-scale feature fusion within the network. As shown in Figure 6, P3, P4, P5, P6, and P7 denote the output layers of the backbone network. Each output layer produces corresponding feature maps, with distinct channel counts and spatial resolutions. Hollow circles represent feature tensors, while solid-colored circles denote feature processing operators. Every connected edge is assigned a learnable weight w. Resolution adjustment operations are applied to all upward and downward connections, corresponding to upsampling or downsampling, respectively.

### 2.4. Model Training and Evaluation Metrics

#### 2.4.1. Evaluation Metrics

Precision (P), Recall (R), F1 score, and mean Average Precision (mAP) were used as evaluation metrics for YOLO series models, as shown in Equations (8)–(10).

(8) $$P=\frac{T_{P}}{T_{P}+F_{P}},$$

(9) $$R=\frac{T_{P}}{T_{P}+F_{N}},$$

(10) $$F1=\frac{2PR}{P+R},$$

where *T_P_* denotes the number of positive samples correctly predicted; *F_P_* denotes the number of negative samples predicted as positive samples; *F_N_* denotes the number of positive samples predicted as negative samples.

mAP is the average value of Average Precision (AP) values for all categories. AP refers to the average precision of a single category. The AP value can reflect the prediction accuracy of each category, and the mAP value is used to reflect the overall accuracy of the model, as shown in Equations (11)–(12).

(11) $$AP=\int_{0}^{1\int dr}Precision\times Recall,$$

(12) $$mAP=\frac{\sum_{j=1}^{S}AP\left(j\right)}{S},$$

where *r* denotes the integration variable, which corresponds to the integral of the product of recall and precision. *S* represents the total number of categories. In this study, detection is carried out for two target categories of maize and weeds; therefore, *S* equals 2.

#### 2.4.2. Training Environment and Parameter Settings

All experiments were performed on a desktop computer equipped with an Intel Xeon w5-3435X processor and an NVIDIA GeForce RTX 4090 graphics card with 24 GB video memory, running a 64-bit Windows operating system. The software environment comprised Python 3.11.15, Ultralytics 8.4.11, PyTorch 2.5.0 with CUDA 11.8 and cuDNN 9.1.0.

The model was trained for 300 epochs with a batch size of 64, referring to the commonly used training configurations in related agricultural object detection studies where YOLO series models are typically trained for 200 to 300 epochs. The input image size was uniformly set to 640 by 640 pixels. The optimizer was stochastic gradient descent with a momentum of 0.937 and a weight decay of 0.0005. The initial learning rate was 0.01, which was linearly decayed to a final value of 0.0001 over the training process, with a warm-up period of 3 epochs. The classification loss was binary cross-entropy, the bounding box regression loss was CIoU, and the distribution focal loss was employed for box distribution learning. No pretrained weights were loaded, and the network was initialized from the yolov12n.yaml architecture configuration file. The random seed was fixed to 0, and deterministic operations were enabled to ensure reproducibility. Early stopping was not enabled, and the best model checkpoint was selected according to the highest fitness score on the validation set. During validation, the confidence threshold was set to 0.001 and the NMS IoU threshold to 0.7. For prediction, the confidence threshold was 0.25, and the maximum number of detections per image was 300.

## 3. Results

### 3.1. Analysis of Dataset Sample Distribution Characteristics

**Figure 7:**
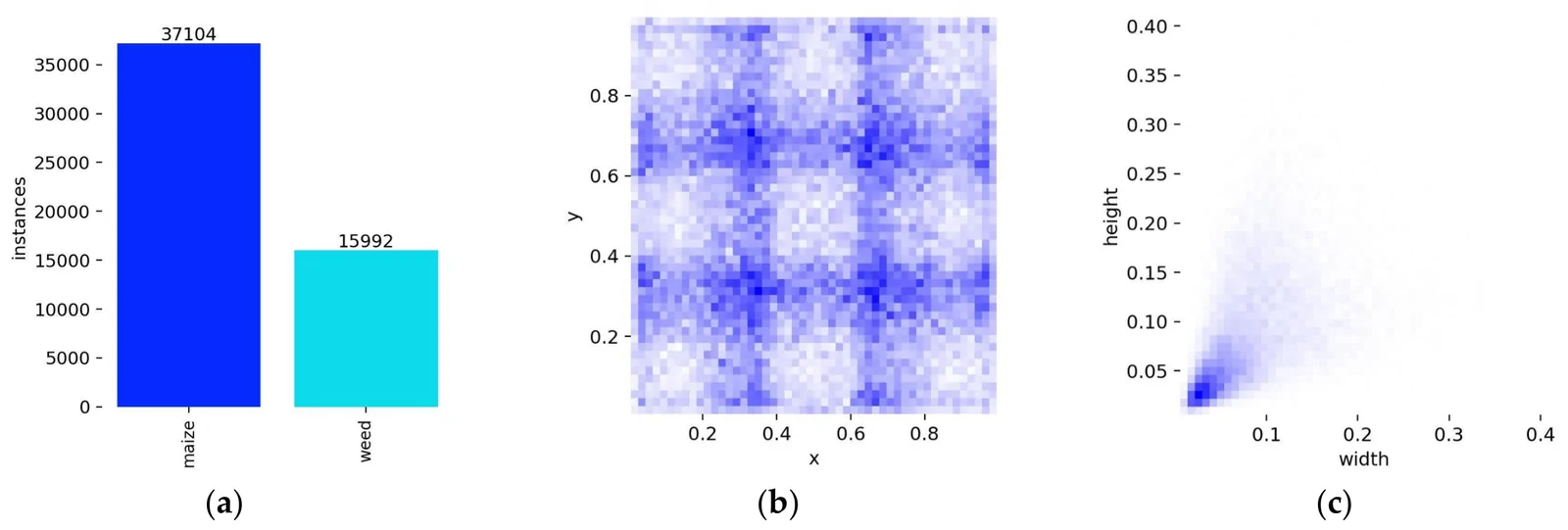
Sample distribution statistics. (**a**) Annotation quantity per category, with n = 37,104 maize and n = 15,992 weed instances; (**b**) Heatmap of annotation box center coordinates, with normalized x and y axes; (**c**) Normalized density distribution of annotation box width and height.

Quantitative statistics and visual analysis are conducted on all annotated samples from the previously described maize seedling stage field image dataset. The sample distribution statistics are presented in Figure 7. The figure contains three subplots, arranged from left to right: the annotation count per category, a heatmap of annotation bounding box center coordinates, and the normalized density distribution of annotation bounding box dimensions.

As shown in Figure 7a, the dataset contains 37,104 annotated maize instances and 15,992 annotated weed instances. This reveals a clear class imbalance across the two categories. This substantial quantity gap may lead to biased network learning weights for the two target classes during model training. As shown in Figure 7b, maize and weed targets are evenly distributed across the normalized horizontal and vertical coordinate ranges of all images, with no obvious clustering at image edges or corners. This indicates no systematic framing bias during field image acquisition, and confirms that the dataset is representative of real farmland scenarios. Figure 7c indicates that the normalized width and height of most maize seedlings and weeds are below 0.1 and 0.05, respectively, meaning fine small-scale field targets account for a large share of the dataset. The default anchor box sizes of the original YOLO series models have poor alignment with these tiny weed targets. This challenge is further amplified by motion blur from handheld image acquisition, which weakens weed edge textures and makes it harder for the model to separate weeds from the soil background.

### 3.2. Comparison of Detection Performance of Different Models

To identify an appropriate baseline detection network for this dataset, five YOLO series lightweight models, including YOLOv8n, YOLOv10n, YOLO11n, YOLOv12n, and YOLO26n, are evaluated under identical comparative experimental conditions. All models use the exact same dataset split, image augmentation pipeline, training hyperparameters, and hardware setup. Four evaluation metrics, namely precision (P), recall (R), F1 score, and mAP@0.5, are used to quantify the recognition performance for maize and weeds separately. The comparison of maize and weed detection performance across different models is summarized in Table 1.

A comprehensive analysis of the results in Table 1 shows that all five models consistently achieve stronger recognition performance for maize than for weeds. For maize, all models reach precision values above 0.85, recall values steadily above 0.86, with F1 scores and mAP@0.5 above 0.86 and 0.90, respectively. By contrast, the maximum weed mAP@0.5 across all baselines is only 0.7328. Weed detection remains a shared limitation of all five baseline models, indicating substantial room for targeted improvement.

**Figure 8:**
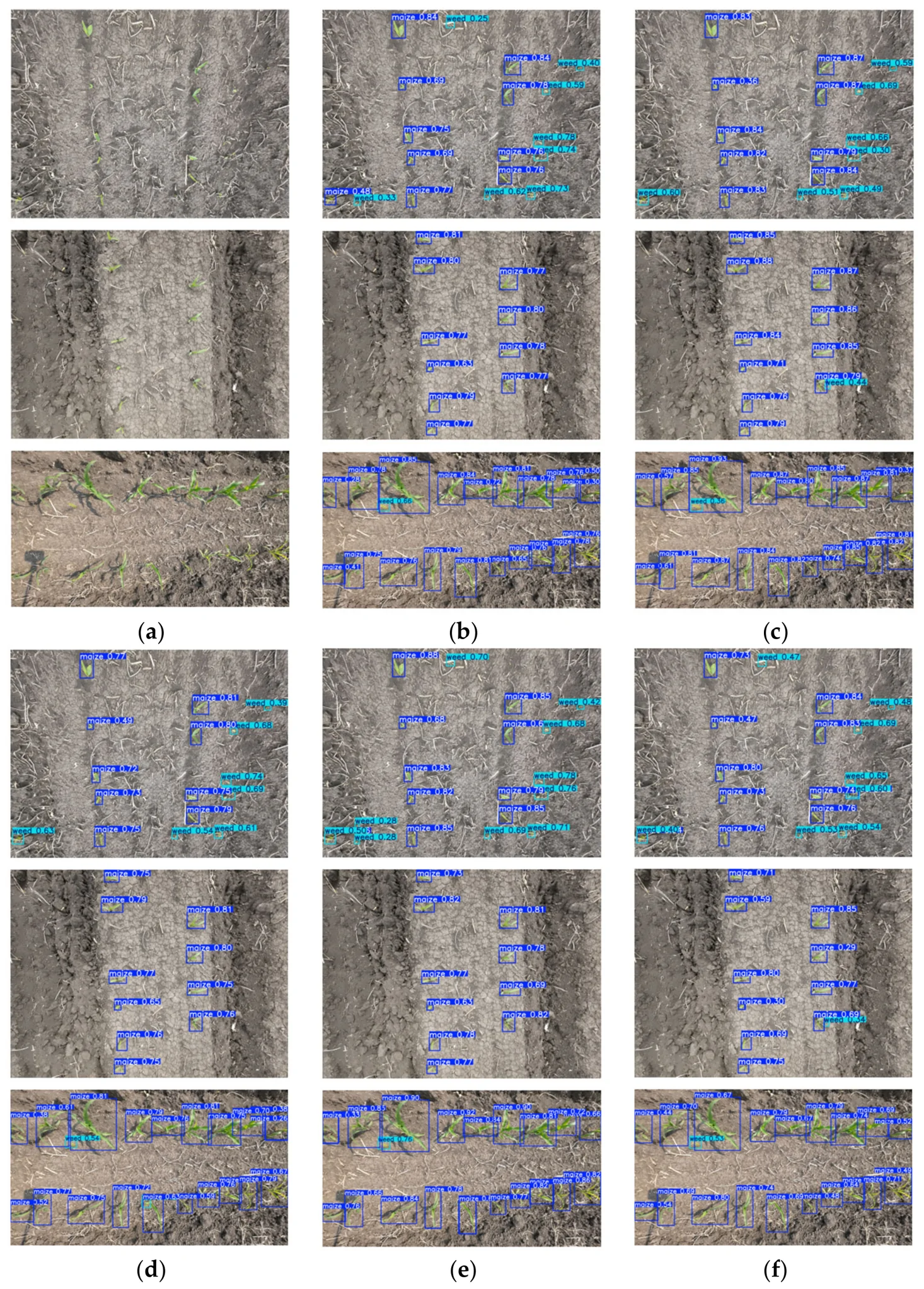
Visual comparison of detection performance across models. (**a**) Original images; (**b**–**f**) results of YOLOv8n, YOLOv10n, YOLO11n, YOLOv12n and YOLO26n. Blue boxes denote detected targets with category labels and confidence scores.

Cross-model comparison of the four weed-focused evaluation metrics shows that YOLOv12n achieves the highest precision of 0.7697, followed in order by YOLO11n, YOLO26n, YOLOv8n, and YOLOv10n. Higher precision reflects a stronger ability to distinguish weeds from soil background and reduce false positive detections. For recall, YOLO11n ranks highest at 0.6420. YOLOv12n and YOLOv8n deliver closely matched recall values, while YOLO26n and YOLOv10n show weaker recall performance. Higher recall corresponds to fewer missed detections of field weeds. For the F1 score, which balances precision and recall, YOLOv12n delivers the best result of 0.6965, followed by YOLO11n, with performance declining sequentially for the remaining models. For mAP@0.5, which reflects overall comprehensive weed detection performance, YOLOv12n again achieves the best result, followed by YOLO11n, YOLOv8n, and YOLO26n. YOLOv10n delivers the weakest comprehensive weed detection performance. To visually illustrate the detection differences across models under real field conditions, three sets of representative field images are selected, covering scenarios with fine sparse weeds, bare soil cracks, and densely mixed crop and weed seedlings. Visual comparison of detection performance across models is presented in Figure 8.

As shown in Figure 8, blue rectangular boxes mark detected targets, with category labels and associated confidence scores displayed above each box. All five models successfully detect maize seedlings across all test images, with no large-scale missed detections. However, clear performance gaps emerge across the models when detecting tiny weeds growing close to the soil surface. YOLOv8n and YOLOv10n show limited recognition ability for fine weeds and fail to generate detection boxes for numerous small targets, leading to obvious missed detections. YOLO11n misses a small number of weeds in complex bare soil regions. YOLO26n detects a larger number of fine weeds, but many of these detections have low confidence, and soil regions with weed-like textures are frequently misclassified as weed targets. YOLOv12n reliably generates detection boxes for fine weeds, with the fewest false positives and missed detections.

The core goal of algorithm improvement in this study is to boost weed recognition accuracy without degrading the already strong maize detection performance. When combining quantitative evaluation metrics and qualitative field detection results, YOLOv12n delivers the best balance between stable maize recognition and baseline weed detection performance. Accordingly, YOLOv12n is selected as the baseline for all subsequent algorithm optimization work.

### 3.3. Analysis of Ablation Experiment Results

#### 3.3.1. Comparative Analysis of Combined Multiple Improved Modules

To quantitatively verify the individual contribution and joint optimization effect of RepViTBlock, MFMSA, and BiFPN, seven improved model variants are constructed on top of the original YOLOv12n baseline, covering single-module, dual-module, and full three-module combinations. All variants use the exact same dataset, training hardware, number of training epochs, and hyperparameter configurations. Maize AP@0.5, weed AP@0.5, and mAP@0.5 across the two categories are taken as quantitative evaluation criteria. All ablation results are evaluated on the held-out test set. The results of the ablation experiment are summarized in Table 2.

As shown in Table 2, compared with the YOLOv12n baseline, the three single module models YOLOv12n-R, YOLOv12n-M and YOLOv12n-B achieve slight increases in both maize AP@0.5 and weed AP@0.5. Among them, YOLOv12n-R with only RepViTBlock obtains the largest improvement in weed AP@0.5 among single-module groups. This indicates that replacing C3k2 with this module enhances the capability to capture fine-grained features of tiny field weeds through the reparameterized feature extraction structure, which effectively alleviates the loss of features for ground-attached weeds. YOLOv12n-M with MFMSA weakens background noise interference from soil and crop residues via multi-frequency and multi-scale feature aggregation, leading to moderate improvements in weed AP@0.5 and overall mAP@0.5. YOLOv12n-B equipped with BiFPN optimizes weight allocation for multi-scale feature fusion and mitigates the problem that shallow weed features are suppressed by large-scale maize features, bringing positive gain to weed AP@0.5.

The three dual-module combined models YOLOv12n-RM, YOLOv12n-RB and YOLOv12n-MB yield higher maize and weed AP@0.5 than all single-module control groups. This demonstrates that the three modules are functionally complementary without performance offset or conflict. YOLOv12n-RB achieves the best mAP@0.5 among dual-module groups, indicating that the combination of backbone feature enhancement and cross-layer weighted fusion brings higher comprehensive benefits for maize and weed detection. YOLOv12n-RMB with the fusion of all three modules obtains the optimal mAP@0.5 metrics across all groups. Compared with the original baseline, its maize AP@0.5 increases from 0.9395 to 0.9745, weed AP@0.5 rises from 0.7328 to 0.7820, and mAP@0.5 improves by 0.0421. Based on all ablation results in Table 2, YOLOv12n-RMB is determined as the optimal detection model in this study.

#### 3.3.2. Analysis of Training Curves for YOLOv12n-RMB

**Figure 9:**
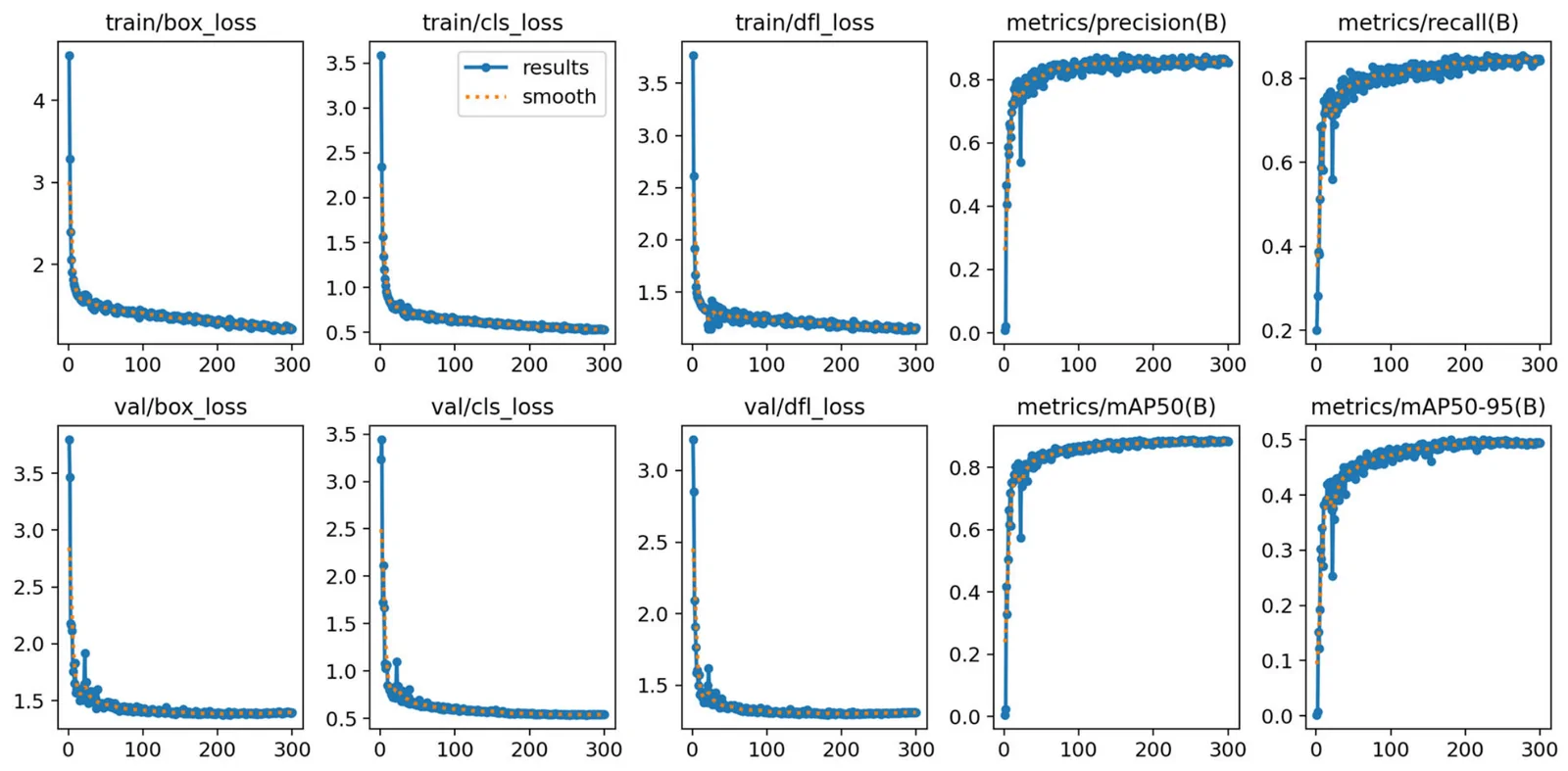
Training curves of YOLOv12n-RMB over 300 epochs, showing training and validation losses (box, classification, and distribution focal loss) as well as validation Precision, Recall, and mAP.

The training curves of YOLOv12n-RMB over 300 epochs are illustrated in Figure 9, showing the trends of training loss, validation loss, Precision, Recall, and mAP with increasing epochs.

As shown in the loss curves, box localization loss, classification loss, and distribution focal loss drop rapidly during the first 100 epochs, then converge gradually and stabilize between epochs 100 and 300. The training and validation loss curves align closely, with no obvious divergence or sharp upward trend in later training stages, indicating that the model does not suffer from overfitting and demonstrates strong generalization performance. Precision, recall, and mAP increase rapidly during the first 100 epochs, with growth slowing noticeably after epoch 200. All metrics reach stable peak values by the end of the 300th epoch. This confirms that the 300-epoch training schedule is appropriate, and that the model has fully learned the discriminative features separating maize and weeds.

#### 3.3.3. Feature Curve Analysis of YOLOv12n-RMB

**Figure 10:**
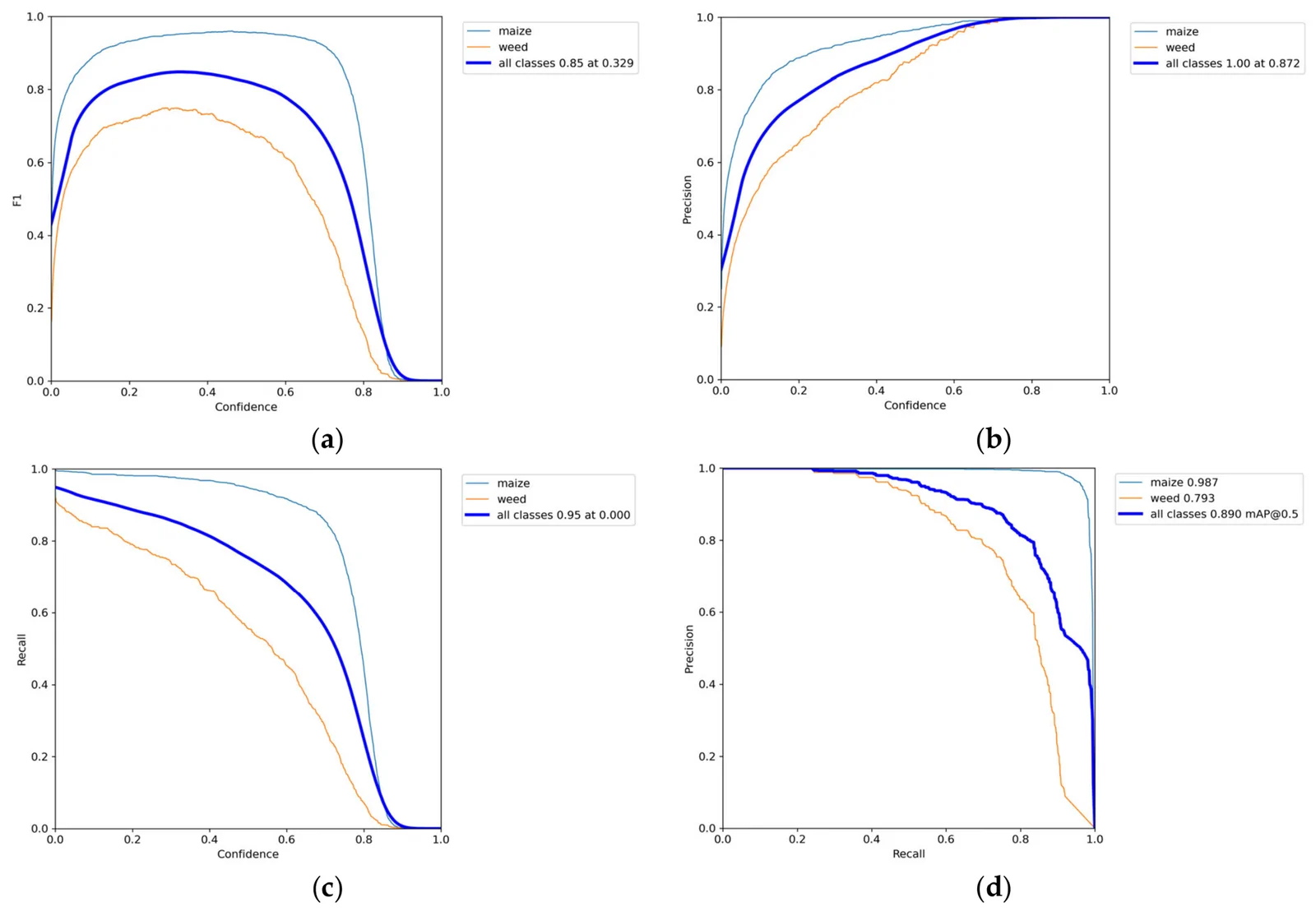
Feature curve of YOLOv12n-RMB. (**a**) F1-Confidence Curve; (**b**) Precision-Confidence Curve; (**c**) Recall-Confidence Curve; (**d**) Precision-Recall Curve.

To comprehensively evaluate the recognition stability of YOLOv12n-RMB for the two target categories, four types of feature curves, including F1-Confidence, Precision-Confidence, Precision-Recall, and Recall-Confidence, are plotted on the validation set during training, and the results are shown in Figure 10. It should be noted that these curves and the associated AP values are computed on the validation set.

As shown in Figure 10a, the peak F1 score for maize reaches nearly 0.98, while the peak F1 score for weeds is approximately 0.85. Both target classes reach optimal precision-recall balance at confidence thresholds between 0.3 and 0.5. Weed performance curves remain consistently below those for maize. This gap stems from inherent scenario constraints, including the tiny size of weed targets and strong background interference present in the dataset. Even so, clear improvements in precision, recall, and F1 score are observed relative to the baseline model. As shown in Figure 10b, precision values for both maize and weeds approach 1 as the confidence threshold rises. Nearly no background false detections remain at high confidence thresholds, which confirms that the combined C3k2-R and A2C2f-M design effectively strengthens the model’s ability to distinguish foreground and background features. As shown in Figure 10c, recall values for both targets rise as the confidence threshold decreases. A notable improvement in weed recall is visible in the low confidence range, which demonstrates that Concat-B weighted fusion effectively reduces missed detections of tiny weeds. As shown in Figure 10d, the AP value of maize reaches 0.987, the AP value of weeds is 0.793, and the overall mAP reaches 0.890 on the validation set. The maize PR curve remains close to the upper axis across the full confidence range, indicating extremely high recognition stability. The weed PR curve dips slightly across the middle confidence segment. This indicates that a small number of extremely small and occluded weeds remain challenging to detect. This constraint directly caps the upper bounds of weed F1 and mAP relative to maize, and represents a remaining limitation of the current model.

#### 3.3.4. Analysis of Normalized Confusion Matrix

**Figure 11:**
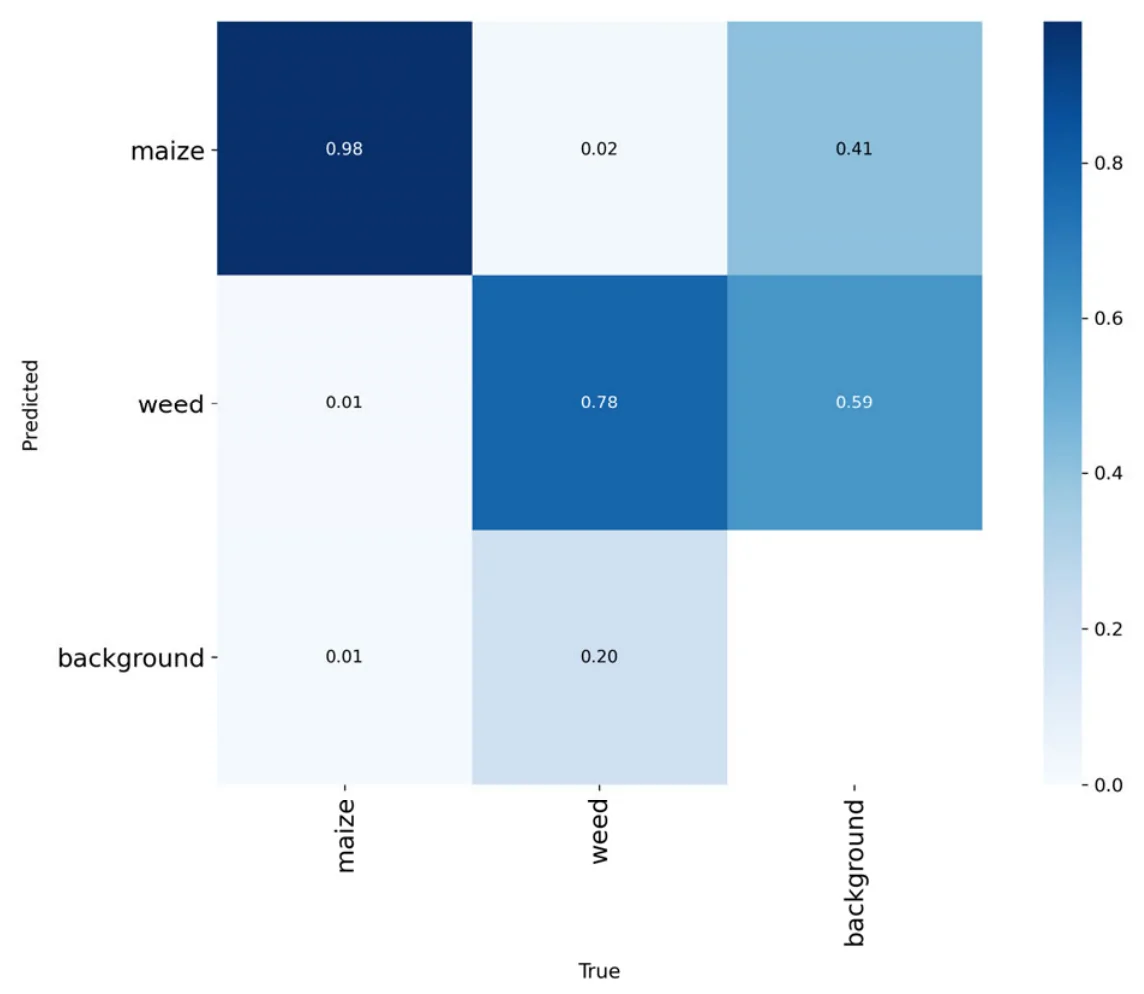
Normalized confusion matrix of YOLOv12n-RMB, with ground truth categories on the horizontal axis and predicted categories on the vertical axis (maize, weed, and background).

The normalized confusion matrix of the proposed YOLOv12n-RMB on the held-out test set is presented in Figure 11. The horizontal axis denotes ground truth sample categories, while the vertical axis denotes model-predicted categories, covering maize, weed, and background classes. For ground truth maize samples, the model achieves a correct prediction rate of 0.98. Only 0.02 of maize samples are misclassified as background or weeds. This translates to high maize recall, consistent with the strong maize F1 and mAP values reported earlier. For ground truth weed samples, the correct recognition rate is 0.78, with 0.20 of weeds misclassified as background, which directly lowers weed recall. A small share of weeds is also confused with maize seedling features. For the background class, soil and crop residues are almost never misclassified as maize, though some background textures are predicted as weeds. This directly reduces weed precision and limits further gains in weed F1 and mAP.

While real-world field conditions, including tiny weed size, partial occlusion, and visual texture similarity, cause a small number of missed and false detections for extremely fine weeds, these limitations stem from inherent properties of the collected dataset and field scenarios and do not undermine the core performance of the model for maize and weed recognition.

### 3.4. Analysis of Field Practical Verification Results

**Figure 12:**
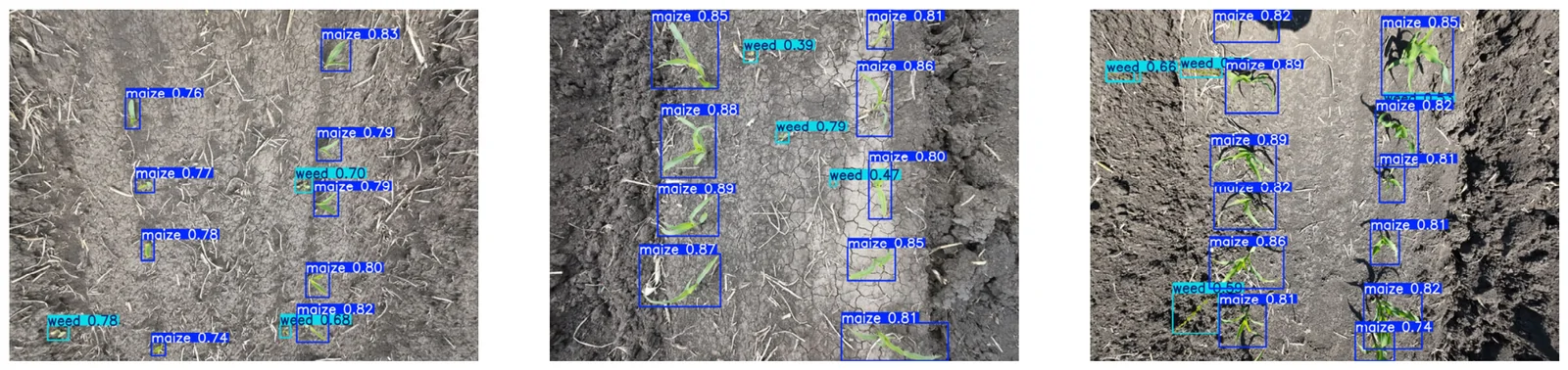
Field detection results of YOLOv12n-RMB. Three representative images (n = 3) selected from an independent verification set of n = 300 images.

After comparing the mAP@0.5 metrics of each model through ablation experiments, YOLOv12n-RMB is determined as the optimal detection model. For practical scenario verification, 300 field images were independently collected on different dates and from different field plots, and these images do not belong to the training, validation, or test sets of the dataset. The verification set covers various working conditions, including diverse illumination, maize plants at different seedling stages, and mixed weeds, to examine the actual recognition capability of the model under real farmland scenarios. Three representative images were selected from the verification set for visualization, and their detection results are presented in Figure 12.

The three representative images cover multiple typical field conditions, including weak illumination, strong direct sunlight, different seedling densities, and scattered weed distribution. Maize seedlings show obvious differences in growth stages, and complex scenarios such as ground-attached fine weeds and weeds overlapping with or growing alongside maize plants are also included, representing the challenging conditions encountered in practical weeding operations. It can be observed from the visualization results that YOLOv12n-RMB can completely and accurately generate bounding boxes for all maize seedlings and weed targets in images regardless of variations in field illumination conditions and maize growth stages, without large-scale missed or false detections. The values inside boxes in the figure represent classification confidence scores output by the model. Some weed targets obtain low confidence scores due to their tiny plant size and high similarity with bare soil textures. Nevertheless, low confidence scores only reflect limited classification certainty for such targets and will not interfere with the generation of target bounding boxes. Accurate bounding box annotation for maize and weed regions can still be achieved.

Comprehensive qualitative results from on-site field trials confirm that YOLOv12n-RMB adapts reliably to a wide range of complex field environments encountered during the maize seedling stage in high-latitude cold regions. The model delivers stable localization and recognition performance for maize under varying illumination conditions and across different seedling growth stages, as well as for a broad range of fine weed targets. Reliable bounding boxes are generated even for low-confidence weed targets, fully meeting the practical on-site visual recognition requirements for maize and weed detection.

## 4. Discussion

### 4.1. Rationale for Baseline Model Selection

Among the five YOLO variants evaluated, YOLOv12n achieved the most balanced trade-off between maize and weed detection, which motivated its selection as the baseline. Although GTDR-YOLOv12 reported strong performance on general agricultural weed datasets [36], the default attention-centric architecture was originally designed for balanced general object categories rather than for herbaceous plants with extreme size variation within a single maize seedling field. Earlier YOLO versions have likewise shown that direct transfer to complex weed scenes does not guarantee sufficient performance, because small target scale, dense plant distribution, and soil background interference collectively degrade feature discriminability [37]. Selecting YOLOv12n as the baseline therefore allows the present study to isolate the contribution of the three proposed modules from the choice of backbone family, rather than confounding architectural differences with the effect of each module.

### 4.2. Independent Contribution and Synergy of the Proposed Modules

The three modules were designed to address distinct bottlenecks rather than overlapping functions, and the ablation results are consistent with this design intent. C3k2-R replaces the default C3k2 block with a reparameterized RepViTBlock structure. Reparameterization-based backbones have been adopted in several recent agricultural detection studies because they strengthen multi-branch feature extraction during training while collapsing to a single branch at inference, avoiding runtime overhead [38,39]. A maize seedling detection study also reported that reparameterized blocks improved small target recognition without increasing model parameters [40]. The present results show that C3k2-R produced the largest single module gain on weed AP@0.5 among the three modules, supporting the view that fine-grained feature extraction is the primary bottleneck for ground-attached tiny weeds.

The MFMSA module introduces multi-frequency, multi-scale attention into the neck. Frequency domain decomposition has been shown to separate low-frequency background patterns from high-frequency object details in crop imagery [33], which is directly relevant to the present field where soil and crop residues occupy most of the image. The A2C2f-M variant therefore acts as a noise suppression mechanism rather than a raw capacity increase. Concat-B replaces the standard top-down fusion path with a bidirectional weighted feature pyramid network. BiFPN has been introduced into several recent weed detection models because it allows the network to assign learnable weights to multi-scale features instead of treating all fusion paths equally [41,42]. A cotton field weed detection study reported that BiFPN improved recognition of weed features in complex backgrounds when combined with a compact backbone [41], which aligns with the present finding that Concat-B produced a positive gain on weed AP@0.5 while preserving maize detection.

When the present model is compared quantitatively with recent maize and weed detection studies, it remains competitive despite using only RGB imagery collected under cold region conditions. The improved YOLOv8n model of Qi et al. reported weed detection accuracy on a Jiangsu maize field dataset, and the BACD-YOLO model of Wang et al. achieved embedded deployment on maize seedling weed detection with a compact architecture [13,14]. The cereal weed detector of another study reported high mAP on a controlled cereal dataset [28]. The present YOLOv12n-RMB model achieves maize AP@0.5 of 0.9745 and weed AP@0.5 of 0.7820 on a cold region maize seedling dataset containing both crop and weed instances with strong background clutter. HDMS-YOLO also reported that multi-scale feature fusion is essential for small weed recall in complex farmland environments [43], which further supports the design of the present model.

### 4.3. Sources of Recognition Error

The confusion matrix shows that the principal residual error is weeds being misclassified as background rather than weeds being confused with maize. This pattern is consistent with the class imbalance observed in the present dataset, where maize outnumbers weeds by a factor of approximately 2.3, and is also consistent with the broader literature. HDMS-YOLO reported that small and visually similar weeds such as goosefoot and poppy exhibit the lowest recall among all categories [43], indicating that minority small object categories remain the hardest detection target even in specialized weed models. Variations in field illumination and background conditions have likewise been reported to cause notable accuracy fluctuations when detection models are evaluated across different field periods [37]. Motion blur introduced by camera movement or wind during handheld acquisition is another plausible contributor to missed weed instances. Motion blur has been identified as a key factor reducing weed recognition in handheld and aerial imagery, and specialized deblurring preprocessing networks have been proposed to restore blurred weed images before detection [44].

Occlusion between neighboring plants further explains the residual false negatives. A recent weed instance segmentation study on soybean fields reported that severe occlusion between weed species caused multiple missed detections, even when the overall segmentation performance was high [45]. The present field images were collected under natural canopy overlap conditions, so a portion of the low-confidence weeds visible in the qualitative results likely corresponds to partially obscured targets. Across all recent weed detection studies, small target recall remains the weakest metric relative to precision and overall mAP [36,37], which indicates that the present weed AP@0.5 of 0.7820 is within the expected range of the current state of the art rather than an outlier.

### 4.4. Limitations and Future Work

Three limitations of the present study should be acknowledged. First, only RGB imagery acquired by a single consumer-grade action camera was used, and no multispectral or near-infrared channels were available. Recent studies have demonstrated that multispectral UAV imagery incorporating near-infrared and red edge bands can improve crop weed discrimination compared with RGB-only imagery [46,47]. Multispectral data provide spectral separation between vegetation and soil that visible bands alone cannot capture, particularly under varying illumination conditions [47]. However, multispectral cameras substantially increase acquisition cost, calibration effort, and data storage requirements, and the present study deliberately focused on the lowest-cost sensor configuration so that the detection pipeline remains accessible to extension users in cold-region maize production.

Second, the present dataset covers maize seedling stages from two to five leaves only, and the weed species present are those dominant in the local cold region climate. Generalization to other weed species, later maize growth stages, and different climatic zones cannot be assumed without additional field data. Expanding the dataset across growth stages, weed species, and geographic regions is therefore a priority.

Third, although the proposed model was evaluated on an independent field image set, no embedded deployment or dynamic inference testing has been conducted. Recent agricultural detection studies have shown that structured pruning and edge-oriented optimization can reduce the computational load of YOLO series models while retaining most of their accuracy on embedded platforms [48]. These compression strategies are therefore natural next steps for the present model before field deployment.

Finally, the present study demonstrates detection but does not yet close the loop with an actuated weeding mechanism. Systematic reviews of intelligent mechanical weed control have concluded that the integration of vision-based detection with variable rate spraying and mechanical uprooting is feasible and represents the dominant engineering direction for next-generation weed management [49]. Future work will therefore integrate the present detector with a variable rate sprayer or mechanical weeding actuator, evaluate dynamic recognition performance in complex scenes, and further improve small weed recall through deblurring preprocessing [44] and multispectral extension.

## 5. Conclusions

This study proposes a YOLOv12n-RMB detection model that integrates multiple modules for weed identification during the maize seedling stage in cold regions. The specific research conclusions are as follows:

(1) Among the five baseline models, YOLOv8n, YOLOv10n, YOLO11n, YOLOv12n, and YOLO26n, YOLOv12n achieves the highest weed precision of 0.7697, the highest weed F1 score of 0.6965, and the highest weed mAP@0.5 of 0.7328, while maintaining maize mAP@0.5 at 0.9395. Its balanced performance on both target categories makes it the most suitable baseline for cold region seedling stage field detection.
(2) The three proposed modules address distinct bottlenecks of the baseline network. C3k2-R strengthens fine-grained feature extraction for tiny weeds, A2C2f-M separates high-frequency weed details from low-frequency soil background through frequency domain decomposition, and Concat-B adaptively balances multi-scale features via bidirectional weighted fusion. Ablation experiments confirm that the fusion of all three modules raises maize AP@0.5 from 0.9395 to 0.9745 and weed AP@0.5 from 0.7328 to 0.7820, corresponding to an mAP@0.5 gain of 0.0421 over the baseline.
(3) Training curves show that box localization loss, classification loss, and distribution loss converge steadily within 300 epochs without overfitting. The normalized confusion matrix records a correct prediction rate of 0.98 for maize and 0.78 for weeds, with 0.20 of weeds misclassified as background. Field verification under strong light, weak light, and mixed seedling ages confirms that the model generates accurate bounding boxes for all maize seedlings and most weeds, including low-confidence tiny targets, indicating reliable practical applicability in complex cold region field environments.
(4) Future work will expand the dataset to cover maize at all growth stages and more weed species, introduce multispectral imaging to enhance small target discrimination, and pursue model pruning and quantization. Dynamic recognition experiments integrated with weeding equipment will be conducted to advance the engineering application value of the model.

## Figures and Tables

**Table 1:** Comparison of maize and weed detection performance across different models.

Model	Class	P	R	F1	mAP@0.5
YOLOv8n	maize	0.8554	0.8866	0.8707	0.9067
	weed	0.7234	0.6356	0.6767	0.7181
YOLOv10n	maize	0.8579	0.8786	0.8681	0.9141
	weed	0.7044	0.5933	0.6441	0.6618
YOLO11n	maize	0.8677	0.8698	0.8687	0.9101
	weed	0.7584	0.6420	0.6954	0.7193
YOLOv12n	maize	0.8835	0.9290	0.9057	0.9395
	weed	0.7697	0.6360	0.6965	0.7328
YOLO26n	maize	0.8622	0.9086	0.8848	0.9352
	weed	0.7491	0.5983	0.6652	0.6839

**Table 2:** Results of ablation experiment.

Model	RepViTBlock	MFMSA	BiFPN	AP@0.5 (Maize)	AP@0.5 (Weed)	mAP@0.5
YOLOv12n	—	—	—	0.9395	0.7328	0.8362
YOLOv12n-R	√	—	—	0.9421	0.7406	0.8414
YOLOv12n-M	—	√	—	0.9407	0.7385	0.8396
YOLOv12n-B	—	—	√	0.9413	0.7392	0.8403
YOLOv12n-RM	√	√	—	0.9548	0.7453	0.8501
YOLOv12n-RB	√	—	√	0.9642	0.7536	0.8589
YOLOv12n-MB	—	√	√	0.9456	0.7447	0.8452
YOLOv12n-RMB	√	√	√	0.9745	0.7820	0.8783

“√” indicates that the corresponding module has been introduced in the model; “—” indicates that the module is not enabled.

## Data Availability

The data presented in this study are available on request from the corresponding: author due to the fact that the data are planned to be used for other independent research projects of the authors in the follow-up and cannot be made publicly available for the time being.
